# CURRICULUM MAPPING AS A STRATEGY TO TEACH DIVERSITY IN GERONTOLOGY PROGRAMS

**DOI:** 10.1093/geroni/igac059.039

**Published:** 2022-12-20

**Authors:** Darren Liu, Betty Burston

**Affiliations:** West Virginia University, Morgantown, West Virginia, United States; University of Nevada Las Vegas, Nevada, Las Vegas, Nevada, United States

## Abstract

Curriculum mapping is a process where instructors, program leaders and other instructional designers come together to prepare learning objectives for students throughout a learning journey. The concepts of diversity may be introduced at all levels of a gerontology curriculum. A curriculum that desires to integrate diversity education is especially imperative as it needs to be built upon a guiding principle of a didactic program within which its individual courses, learning objectives, and assessments all together serve the same purpose. This presentation provides insight to the curriculum mappings for diversity education in gerontology programs. While the current programs introduce diversity topics such as race, racism, implicit bias, etc., how and when each topic being introduced in bringing about maximum learning outcomes may be related to a well thought out curriculum mapping. Specifically, this session will go over a few examples of methods for the mappings of diversity topics in different courses.

